# Clinical decision making in cancer care: a review of current and future roles of patient age

**DOI:** 10.1186/s12885-018-4456-9

**Published:** 2018-05-09

**Authors:** Eirik Joakim Tranvåg, Ole Frithjof Norheim, Trygve Ottersen

**Affiliations:** 1 Department of Global Public Health and Primary Care, University of Bergen, Bergen, Norway; 2 Centre for Cancer Biomarkers CCBIO, Department of Clinical Medicine, University of Bergen, Bergen, Norway; 3 Oslo Group on Global Health Policy, Department of Community Medicine and Global Health and Centre for Global Health, University of Oslo, Oslo, Norway; 4 Division for Health Services, Norwegian Institute of Public Health, Oslo, Norway

**Keywords:** Decision making, Clinical practice, Age, Age factors, Personalized medicine, Oncology, Priority setting

## Abstract

**Background:**

Patient age is among the most controversial patient characteristics in clinical decision making. In personalized cancer medicine it is important to understand how individual characteristics do affect practice and how to appropriately incorporate such factors into decision making. Some argue that using age in decision making is unethical, and how patient age should guide cancer care is unsettled. This article provides an overview of the use of age in clinical decision making and discusses how age can be relevant in the context of personalized medicine.

**Methods:**

We conducted a scoping review, searching Pubmed for English references published between 1985 and May 2017. References concerning cancer, with patients above the age of 18 and that discussed age in relation to diagnostic or treatment decisions were included. References that were non-medical or concerning patients below the age of 18, and references that were case reports, ongoing studies or opinion pieces were excluded. Additional references were collected through snowballing and from selected reports, guidelines and articles.

**Results:**

Three hundred and forty-seven relevant references were identified. Patient age can have many and diverse roles in clinical decision making: Contextual roles linked to access (age influences how fast patients are referred to specialized care) and incidence (association between increasing age and increasing incidence rates for cancer); patient-relevant roles linked to physiology (age-related changes in drug metabolism) and comorbidity (association between increasing age and increasing number of comorbidities); and roles related to interventions, such as treatment (older patients receive substandard care) and outcome (survival varies by age).

**Conclusions:**

Patient age is integrated into cancer care decision making in a range of ways that makes it difficult to claim age-neutrality. Acknowledging this and being more transparent about the use of age in decision making are likely to promote better clinical decisions, irrespective of one’s normative viewpoint. This overview also provides a starting point for future discussions on the appropriate role of age in cancer care decision making, which we see as crucial for harnessing the full potential of personalized medicine.

**Electronic supplementary material:**

The online version of this article (10.1186/s12885-018-4456-9) contains supplementary material, which is available to authorized users.

## Background

Among the many patient characteristics that can affect decision making, patient age is both widely used and heavily discussed. Using age appears intuitive in many settings, but exactly how it should guide clinical decisions is unsettled. Incorporating patient age into decision making is by some seen as unethical and discriminatory. Surveys demonstrate that oncologists use patient age when recommending treatment, even when a large majority at the same time state that they are against such use [1, 2]. Among the public, empirical studies demonstrate no consensus on the appropriate role of age when allocating resources [3, 4], although a recent systematic review demonstrated that the public generally favors the young over the elderly when having to give priority to one of the groups [5]. Theoretical arguments are used both for [6, 7] and against [8] the relevance of age as a criteria when allocating resources.

To our knowledge there exists no overview of the role of patient age in clinical decision making in cancer care. Most studies describe age in association with some pre-defined outcome, like treatment selection, survival or shared decision making. A broader examination of how age can influence decision making will benefit both clinical practice and ethical discussion, irrespective of one’s view on the proper role of age. If the use of age is considered unacceptable, it is imperative to identify *all* the ways age actually makes an influence. If every use of age in decision making is discriminatory, every such use of age should be mapped. Equally, if age in some ways can be accepted as guidance for decision making, it is important to know how and to what extent.

With the progress of personalized medicine, attention to individual characteristics will be stronger. In oncology practice it will be increasingly important to understand how patient characteristics affect cancer biology, treatment efficacy, and tolerance [9], as will appropriately incorporating such factors into decision making.

The aim of this study is to provide an overview of the many different ways patient age may guide clinical decisions in oncology. We will identify and discuss associations between age and clinical decisions, and explore how age may be relevant for decision making in the context of personalized medicine.

## Methods

We conducted a scoping review [10] that identified literature covering the use of patient age in cancer diagnostic and treatment decisions. A scoping review is to some extent similar to a systematic review, but there are also several fundamental differences. Systematic reviews address well-defined research questions that can be answered by established methods, and use in-depth assessments of the quality of included studies. Scoping reviews address broader research questions, and can be used to map key concepts of research areas, identify gaps in existing knowledge or merely identify relevant literature on a topic. A scooping review is therefore appropriate to map the many ways patient age may guide clinical decisions in oncology. Scoping reviews do not always assess the quality of included studies, and the synthesis of evidence is typically not quantitative, as it is in systematic reviews [10–12].

We pre-defined our search objective, inclusion criteria and method according to scoping review standards [12]. We searched Pubmed January 21 2016 by combining search terms related to cancer, age and decision making as follows: “(cancer[title] OR “neoplasms”[MeSH Terms]) AND (“age”[Title] OR “age factors”[Mesh]) AND (“decision making”[MeSH Terms] OR decision making[Title/abstract])”, and limited to references published after 1985. References concerning cancer, with patients above the age of 18 and that discussed age in relation to diagnostic or treatment decisions were included. To include newly published research, we did an updated search May 15 2017. We collected additional references through snowballing and from selected reports, guidelines and previously identified articles.

Duplicates were removed and missing abstracts retrieved. Then the abstracts were screened, and references that fulfilled our aim were included. We applied the following exclusion criteria: age under 18 (as we acknowledge that pediatric oncology is a distinct field of medicine), not medically oriented (as the decisions are not taken by physicians), comments and editorials (as they are opinion pieces) and case reports, preliminary findings and ongoing studies (as they are incomplete). Due to the large number of references identified we do not cite them all. Details on all identified articles, including publication year, country, type and keywords on content were gathered in a table and are available in the Additional file 1: Appendix.

Using the chartered details from all references, we analyzed the content of each reference and identified a main topic. We then organized the references based on the topic under three main categories: Context, Patient, and Intervention. This grouping was done after the search, partly in order to organize our findings, and partly to structure and present it in a clinically relevant and informative manner. If a reference fit more than one category, the one best describing the overall aim of the reference was selected. A narrative summary with selected examples from our search describes findings and how they relate to our objectives.

## Results

**Fig. 1:**
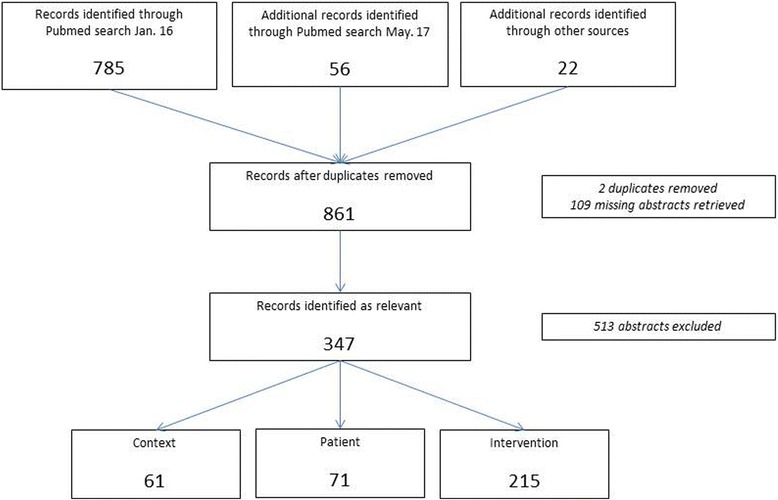
The flow of information through our scoping review

Eight hundred sixty three references were identified (see Fig. 1), including both original research and review articles. After removing duplicates, 861 abstracts were screened using the pre-defined criteria. Of the 347 references identified as relevant, 61 were categorized in the Context group, 71 in the Patient group and 215 in the Intervention group.

Our main finding is that age is associated with and partly influences clinical decisions in ways that are both avoidable, as for access to care (age influences how quickly patients are referred to specialized care) or participation in research (older patients are often underrepresented in clinical trials), and unavoidable, as for incidence (strong association between increasing age and increasing incidence rates for cancer) or comorbidity (association between increasing age and increasing number of comorbidities) or treatment outcomes (decreased survival for older patients). In total these publications show that patient age can be used – directly or indirectly and consciously or unconsciously – to guide decisions (see Table 1).

**Table 1:** Summary of main findings, with examples

Category	Factor	Example
Context	Access	Age influences how fast patients are referred to specialized care
	Incidence	Strong association between increasing age and increasing incidence rates for cancer
	Research	Participants in clinical trials often younger than actual disease population
	Screening	Strict age cut-offs for inclusion in public screening programs
	Guidelines	Clinical guidelines use age thresholds when recommending treatment
Patient	Physiology	Age-related declines in CYP enzymes responsible for hepatic drug metabolism
	Tumor biology	Proportion of ER and HER2 status in breast cancer varies between age groups
	Comorbidity	Association between increasing age and increasing number of comorbidities
	Receptivity	Physicians’ recommendations are more influential for older patients
Intervention	Quality	Older patients tend to receive substandard treatment
	Prediction	Risk prediction tools use age for estimations
	Treatment outcome	High age is often a predictor of decreased survival

### Context

We identified 61 relevant articles associating patient age with factors relevant for the context of a clinical decision. Patient age can influence access to diagnostics and treatment, incidence of cancer, clinical trials and evidence, screening and guideline content.

Access to diagnostics and treatment can be heavily influenced by patient age. Young and old-aged patients recognize fewer cancer symptoms, compared to those aged between 55 and 74 years [13]. And according to the same study by Niksic et al., the number of barriers to present symptoms to a physician decreases with increasing age. When examined, age can influence how fast the patient is referred to further investigation and/or specialist care [14]. Older patients with advanced cancers are less likely to be referred to oncology teams [15] compared to younger patients. And when in specialized care, age can influence the decision to refer to certain types of treatment [16].

There is a well-established link between increasing age and increasing incidence rates for cancer worldwide [17]. In Norway, more than 90% of cancers in men and 85% in women are diagnosed above the age of 50, with almost half of the men and 45% of the women being 70 years or older [18].

Clinical trials are often skewed towards younger and healthier populations compared to the disease population [19], making evidence used in clinical decision weaker. Patients in clinical trials have been shown to be almost 10 years younger than the corresponding Medicare cohort [20]. In the same study, it was demonstrated that studies tend to overestimate survival for older Medicare patients. A systematic review from Zulman et al. shows that one of five trials excludes patients over a certain age, and that almost half of the remaining trials use criteria that disproportionally can exclude older adults [21]. It also found that just one in six trials differentiates benefit by age.

Guidelines for screening use age cut-offs when recommending start and cessation. These are based on estimates of risk, benefit and harm, all of which are influenced by age [22, 23]. Age can also affect the individual patient or physician's decision to screen. Younger women are more likely to be screened for breast or cervical cancer compared to older women [24, 25], and general practitioners’ tendency to screen for prostate cancer using PSA-tests increase with increasing patient age [26].

Several treatment guidelines use age in their recommendations. Some use age when recommending treatment type and length, like the new ESMO guideline on treatment of metastatic non-small cell lung cancer which explicitly emphasizes the age of 70 [27]. The ESMO guideline for treatment of acute lymphoblastic leukemia uses age-adapted treatment protocols in their treatment recommendations [28]. Age can also be listed as one relevant factor for deciding treatment [29], and it can be used as guidance when referring patients to further diagnostics when suspicious of cancer disease [30]. NICE uses age as an explicit cut off when deciding the cost-effectiveness of genetic testing for individuals with a family history of breast cancer [31].

### Patient

Seventy-one relevant articles associate patient age with relevant patient factors in clinical decision making. Comorbidity, physiology, tumor biology and patient receptivity for information and communication are all associated with patient age.

A review by Pal and Hurria report that age-related decline in renal blood flow and glomerular filtration rate may affect clearance of cytotoxic agents [9]. Liver size and blood flow decrease by age, and so does effects of many CYP enzymes responsible for hepatic drug metabolism [32]. Compared to younger patients, older patients have reduced stem cell reserve, reduced reserve of functional tissue, and increased risk of comorbidity and polypharmacy [33].

There is a solid link between increasing age and prevalence of comorbidity [34]. In a large observation of newly diagnosed cancer patients, both severity and the mean number of comorbidity conditions increased by age [35]. Findings in a systematic review by Lee et al. suggest that cancer patients with comorbidity receive less chemotherapy and have inferior survival compared to patients without comorbidity [36].

Age is often linked to certain cancer biology and molecular pathology patterns. In breast cancer, medullary and inflammatory disease types are more common in younger patients, while papillary, lobular and mucinous types are more common in older patients [37]. Patients under 45 years of age have almost double the proportion of ER-/HER2- tumors and half the proportion of luminal A tumors than patients above 65 years [38]. Similar age-associated pathology patterns are seen in other cancer types [39, 40].

Age can affect patient’s information processing and participation in decision, requiring physicians to adjust their communication and decision style. There is robust evidence of age-related decline in deliberative functions [41], which suggests that information given is processed more slowly. Older patients also tend to make more immediate treatment decisions, with one hypothesis being more limited cognitive resources [42]. A recent systematic review suggests that physicians’ recommendation is more influential for older patients [43]. Age is also shown to influence information need: younger patients below the age of 55 require more information than older patients [44].

### Intervention

We identified 215 relevant articles grouped under the broad term interventions. More than half of the references (125) relate patient age to treatment outcome, while others associate age with other relevant factors like prediction tools and quality of treatment.

The outcome of cancer is influenced by the age of the patient, with decreasing survival for older patients [18, 39, 40, 45, 46]. For many cancer types, high age is a predictor of mortality [47–49]. However, this does not apply exclusively for older patients: Fredholm et al. have shown that women with breast cancer under the age of 35 have distinctly worse survival, even with higher intensity treatment [50].

Register studies show that older patients tend to receive substandard treatment: the proportion of lung cancer patients receiving guideline treatment declines with increasing age [51]. Older patients with colorectal cancer were less likely to receive the new anti-angiogenetic drug bevacizumab [52]. Patient age is a significant predictor of type of breast cancer surgery. Younger women receive breast conservation surgery more often than older women [53]. Backing this are many surveys, reporting that physicians do take patient age into consideration when deciding cancer treatment [54–57].

There are many different risk prediction models in use for estimation of survival. One of the best known, Adjuvant! Online(AO) incorporates patient age as a factor [58]. It is shown that AO overestimates survival in both the younger (below 40 years) and oldest (above 75 years) age groups [59, 60]. Other prediction tools that are used in oncology also include age, like Predict [61], for deciding treatment after breast cancer surgery, and a new model for predictions of chemotherapy toxicity, developed by Hurria et al. [62].

## Discussion

This scoping review is to our knowledge the first attempt to methodically map out the role of patient age in clinical decision making in cancer care. Our findings suggest that patient age is widely used, directly or indirectly and consciously or unconsciously, to guide clinical decisions.

Patient age is integrated into clinical decision making in a range of ways that in sum makes it not only difficult, but almost meaningless to claim age-neutrality. Consequentially, beliefs that physicians do and even can make decisions completely independent of patient age should be discarded, as such beliefs probably hinders due consideration and discussion of the role of age. Denying any role of age is thus unproductive and can be harmful both for patients and for the debate. Instead, it is time to critically appraise how much and in which ways patient age should guide clinical decisions.

Accepting the relevance of patient age is important in a clinical setting. A more transparent discussion will make clinicians more attentive to their own decision making strategy, thereby facilitating fair and consistent decisions. The opposite, an intentional or unintentional neglect of patient age, is likely to result in poor decisions. In particular, it may lead to unjustified age-based discrimination, in the sense that decisions based on age are not systematically considered or justified. Acknowledging the complex role of age in clinical decision making will also benefit the academic debate. Research is often framed as yes–no decisions on the direct influence of age [3, 4], while our findings demonstrate a variety of possible ways age influences clinical decisions.

Deciding when and how patient age can be justified is a value judgement. In some cases, it is unproblematic. Few, if any, will argue that taking into account the well-documented association between increasing age and increasing incidence of cancer is discriminatory. Nor is anyone protesting that communication between patients and physicians should be adapted to the patient’s age and mental status. In these cases the use of patient age is uncontroversial. Conversely, the poor representation of older patients in clinical trial populations needs to be addressed.

Often decisions about individual patients are based on group level data, and age is typically used indirectly as a proxy for individual patient characteristics. In modern cancer care this practice will increasingly be replaced by biomarkers or composite measures. Pharmacodynamic biomarkers can inform the optimal drug dosage for a patient better then estimates based on age [63]. New cancer treatments will increasingly be guided by individual tumor characteristics (see e.g. the Food and Drug Administration’s May 2017 approval of pembrolizumab for any solid tumors with specific genetic features [64]). Comprehensive geriatric assessments will better estimate older patients’ capacity and tolerance of treatment [65]. And biological age can be estimated through various algorithms providing a better description of a patient’s overall mental and physical capacity [66].

For other relationships between patient age and decision making is it more difficult to assess implications for clinical cancer care. Is it a fact, like our review suggest, that older patients receive less and inferior cancer treatment compared to younger patients? Is this true also for new treatments like immunotherapy? If so, is this ethically justifiable? Do oncologists think it is ethically acceptable to limit treatment based on patient age? These questions are important in order to harness the full potential of personalized medicine and require more research. Both empirical and theoretical work is needed.

There are limitations to our study. We have only investigated factors guiding physician recommendations. We acknowledge that deciding treatment is a shared decision between patient and physician, but we still find it valuable to separately investigate these factors. A scoping review does not evaluate the quality of the studies, as is done in systematic reviews. Nevertheless, a scoping review can effectively help identify the many ways age *can* influence decision making – not claiming that age always affects all factors in the same way all the time. A scoping review like this one can also serve as a valuable basis for future in-depth research on influencing factors.

## Conclusion

This article has demonstrated how patient age appears to influence a clinical decision in a variety of ways. While arbitrary use of age can lead to unjustified discrimination, the findings suggest that is difficult, if not impossible for a clinician to make an age-neutral decision. Acknowledging the many roles of age and being more transparent about its use can help clinicians make better and more ethical decisions. It can also promote a more open and informed public debate.

## Additional file

Additional file 1: Appendix Details on identified references. Full references, year of publication, country of publication, type of article, subject of article and type of cancer investigated. (XLSX 45 kb)

## Abbreviations

CYP: Cytochromes P450
ER: Estrogen Receptor
ESMO: European Society for Medical Oncology
HER2: Human Epidermal Growth Factor Receptor 2
NICE: The National Institute for Health and Care Excellence
PSA: Prostate-Specific Antigen
